# Supplementary material for: Cytotoxic Tph subset with low B-cell helper functions and its involvement in systemic lupus erythematosus
Source: Commun Biol. 2024 Mar 6;7:277. doi: 10.1038/s42003-024-05989-x (PMC10918188; doi:10.1038/s42003-024-05989-x)
Supplement: Supplementary file 2 — Supplementary information [file 42003_2024_5989_MOESM2_ESM.pdf]

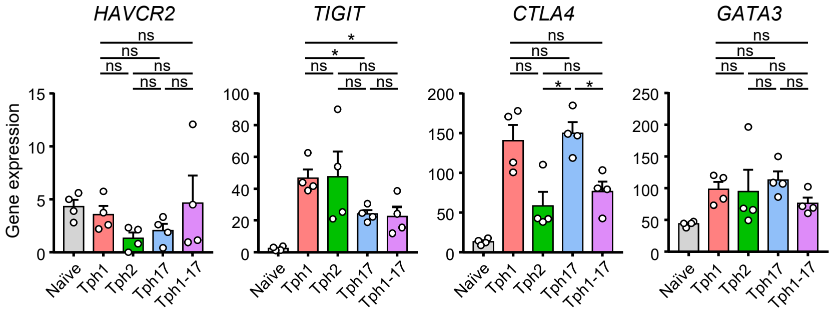

Supplementary Figure 1. The gene expression of *HAVCR2, TIGIT, CTLA4,* and *GATA3* in each Tph subset.

Data represent the mean ± SEM of 4 independent donors.
Data were statistically analyzed using unpaired *t*-test. **P*<0.05, ns: not significant.

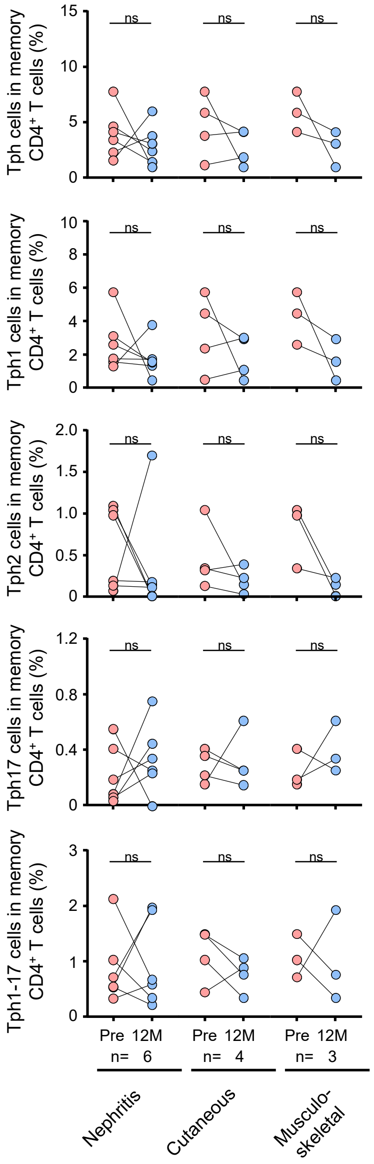

Supplementary Figure 2. The alterations of each Tph subset in case of patients who showed inadequate response for the medications.

Proportions of each Tph subset before and 12 months later in SLE patients who showed inadequate response for the medications with nephritis (n=5), cutaneous (n=4), and musculoskeletal manifestation (n=3). Data were analyzed by Wilcoxon’s signed-rank test. ns: not significant.

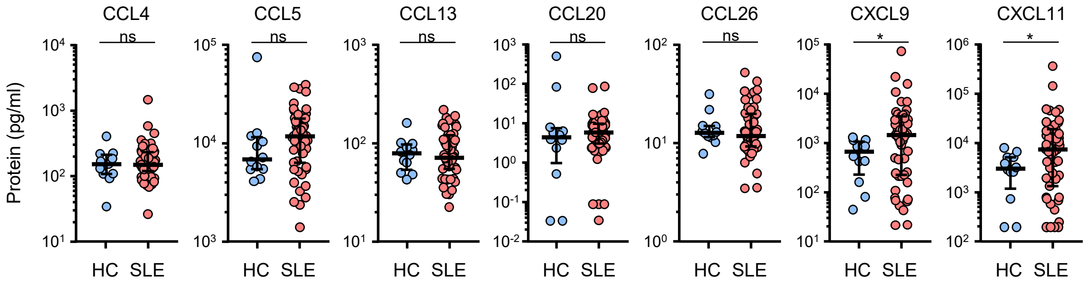

Supplementary Figure 3. The levels of chemokine in the serum.

The levels of CCL4, CCL5, CCL13, CCL20, CCL26, CXCL9, and CXCL11 in the serum of HC (n=12) or SLE patients (n=51). Data represent the median ± IQR. Data were statistically analyzed using Mann-Whitney *U*-test. *P<0.05, ns: not significant.

Supplementary Table 1. The levels of *PDCD1*, *CXCR5*, *CXCR3*, and *CCR6* in naïve CD4⁺ T cells or Tph subsets.

Cells	Gene expression (TPM, mean ± SEM)			
	PDCD1	*CXCR5*	*CXCR3*	*CCR6*
Naïve $CD4^+$ T	0.41 ± 0.24	1.78 ± 0.34	3.14 ± 0.95	0.032 ± 0.032
Tph1	75.62 ± 0.24 ****	3.96 ± 1.76 ns	164.2 ± 20.22 ***	0.41 ± 0.24 ns
Tph2	73.74 ± 8.04 ****	2.05 ± 0.88 ns	24.10 ± 11.55 ns	0 ± 0 ns
Tph17	51.47 ± 5.76 ***	1.84 ± 0.72 ns	5.03 ± 1.49 ns	20.64 ± 2.68 ***
Tph1-17	29.97 ± 4.79 ***	1.72 ± 1.33 ns	133.07 ± 18.54 ***	5.07 ± 0.17 *

Data represent the mean ± SEM of gene expressions in each cell populations (*n*=4).
Statistical differences between naïve $CD4^+$ T cells and Tph subsets were analyzed by unpaired *t*-test.
*P<0.05, **P<0.01, ***P<0.001, ****P<0.0001, ns: not significant.
TPM, transcripts per million

Supplementary Table 2. Selected 357 genes of immunoregulatory molecules for characterization of Tph subsets.

Interleukins / Interleukin receptors		Interferons / Other cytokines	TNF superfamily Members / TNF receptor superfamily members	Chemokines / Chemokine receptors		Tph-related molecules		Cytotoxic molecules
IL1A	IL1R1	IFNA1	LTA	CCL1	CCR1	PDCD1	S1PR1	GZMA
IL1B	IL1R2	IFNA2	TNF	CCL2	CCR2	CD274	S1PR2	GZMB
IL1RN	IL1RAP	IFNA4	LTB	CCL3	CCR3	PDCD1LG	S1PR3	GZMH
IL2	IL2RA	IFNA5	TNFSF4	CCL3L3	CCR4	ICOS	S1PR4	GZMK
IL3	IL2RB	IFNA6	CD40LG	CCL4	CCR5	ICOSLG	S1PR5	GZMM
IL4	IL2RG	IFNA7	FASLG	CCL4L2	CCR6	SH2D1A	SUCNR1	PRF1
IL5	IL3RA	IFNA8	CD70	CCL5	CCR7	CD200	FCRL1	GNLY
IL6	CSF2RB	IFNA10	TNFSF8	CCL7	CCR8	BATF	FCRL2	RUNX1
IL7	IL4R	IFNA13	TNFSF9	CCL8	CCR9	TOX2	FCRL3	RUNX2
IL9	IL5RA	IFNA14	TNFSF10	CCL11	CCR10	TIGIT	FCRL4	RUNX3
IL10	IL6R	IFNA16	TNFSF11	CCL13	CCRL2	NFATC1	FCRL5	NKG7
IL11	IL6ST	IFNA17	TNFSF12	CCL14	CXCR1	CTSB	FCRL6	ZNF683
IL12A	IL7R	IFNA21	TNFSF12	CCL15	CXCR2	CTSW	FCRLA	TCF7
IL12B	IL9R	IFNB1	-TNFSF13	CCL15	CXCR3	CDK5R1	FCRLB	BACH2
IL13	IL10RA	IFNE	TNFSF13	-CCL14	CXCR4	FYN	CCNA2	SPON2
IL15	IL10RB	IFNK	TNFSF13B	CCL16	CXCR5	BTLA	CCNB1	KLRG1
IL16	IL11RA	IFNW1	TNFSF14	CCL17	CXCR6	TBX21	CCNB2	PRSS23
IL17A	IL12RB1	IFNG	TNFSF15	CCL18	ACKR1	EOMES	CCNB3	CRTAM
IL17B	IL12RB2	IFNL1	TNFSF18	CCL19	ACKR2	GATA3	CCNC	CD28
IL17C	IL13RA1	IFNL2	TNFRSF1A	CCL20	ACKR3	RORC	CCND1	PFN1
IL17D	IL13RA2	IFNL3	TNFRSF1B	CCL21	ACKR4	FOXP3	CCND2	EFHD2
IL17F	IL15RA	IFNL4	LTBR	CCL22	CX3CR1	BCL6	CCND3	VCL
IL18	IL17RA	IFNAR1	TNFRSF4	CCL23	XCR1	PRDM1	CCNE1	DIP2A
IL19	IL17RB	IFNAR2	CD40	CCL24		SOX4	CCNE2	SYNE1
IL20	IL17RC	IFNGR1	FAS	CCL25		SLAMF1	CDK1	PLEK
IL21	IL17RD	IFNGR2	TNFRSF6B	CCL26		CD48	E2F1	LPAR1
IL22	IL17RE	IFNLR1	CD27	CCL27		LY9	E2F2	LPAR2
IL23A	IL17REL	CSF1	TNFRSF8	CCL28		CD244	MAF	LPAR3
IL24	IL18BP	CSF2	TNFRSF9	CXCL1		CD84	IRF1	LPAR4
IL25	IL18R1	CSF3	TNFRSF10A	CXCL2		SLAMF6	IRF2	LPAR5
IL26	IL18RAP	CSF1R	TNFRSF10B	CXCL3		SLAMF7	IRF3	LPAR6
IL27	IL20RA	CSF2RA	TNFRSF10C	PF4		SLAMF8	IRF4	LYPLA1
EBI3	IL20RB	CSF3R	TNFRSF10D	CXCL5		SLAMF9	IRF5	ZEB2
IL31	IL21R	TSLP	TNFRSF11A	CXCL6		GPR34	IRF6	ADGRG1
IL32	IL22RA1	CRLF1	TNFRSF11B	PPBP		P2RY10	IRF7	CST7
IL33	IL22RA2	CRLF2	TNFRSF12A	CXCL8		GPR174	IRF8	ZBTB7B
IL34	IL23R	CRLF3	TNFRSF13B	CXCL9		GPR183	IRF9	FGFBP2
IL36A	IL27RA	OSM	TNFRSF13C	CXCL10			IFI44	KLRB1
IL36B	IL31RA	OSMR	TNFRSF14	CXCL11				PTGDR2
IL36G	IL1RAPL1	LIF	TNFRSF17	CXCL12				NCAM1
IL36RN	IL1RAPL2	LIFR	TNFRSF18	CXCL13				
IL37	IL1RL1		TNFRSF19	CXCL14				
IL1F10	IL1RL2		RELT	CXCL16				
C17orf99			TNFRSF21	CXCL17				
METRNL			TNFRSF25	CX3CL1				
				XCL1				
				XCL2				

Supplementary Table 3. Clinical characteristics of SLE patients in this study.

Clinical characteristic	HC (*n*=33)	SLE			
		All (*n*=85)	Inactive (*n*=29)	Active (*n*=56)	New-onset (*n*=27)
Age (years), median (IQR)	40.0 (29.0-50.0)	46.0 (34.0-60.0)	48.0 (37.0-62.0)	43.5 (33.8-58.0)	43.0 (34.0-65.5)
Female, *n* (%)	21 (63.6)	75 (88.2)	26 (89.7)	49 (87.5)	23 (85.2)
Disease duration (years), median (IQR)	—	5.3 (0.1-16.5)	10.2 (5.9-25.9)	1.6 (0.0-10.9)	0.0 (0.0-0.1)
SLEDAI, median (IQR)	—	6.0 (2.0-20.0)	0.0 (0.0-2.0)	17.0 (6.0-22.0)	21.0 (6.5-24.5)
Anti-ds-DNA Ab (IU/ml), median (IQR)	—	17.2 (4.4-74.9)	3.3 (1.8-15.2)	39.6 (10.3-158.8)	49.9 (17.2-260.0)
Classification of renal pathology, *n* (%)	—				
I/II		1 (1.2)	1 (3.4)	0 (0.0)	0 (0.0)
III/IV		29 (34.1)	11 (37.9)	18 (32.1)	7 (25.9)
III/IV+V		7 (8.2)	2 (6.9)	5 (8.9)	0 (0.0)
V		7 (8.2)	3 (10.3)	4 (7.1)	1 (3.7)
White blood cells ($\times 10^3/\mu l$), median (IQR)	3.3-8.6 [a]	4.00 (2.90-5.65)	5.20 (3.95-6.05)	3.50 (2.43-5.10)	3.50 (2.30-5.10)
Lymphocytes ($\times 10^3/\mu l$), median (IQR)	1.56 (1.03-1.92)	0.65 (0.48-0.86)	0.80 (0.73-1.07)	0.52 (0.42-0.80)	0.51 (0.43-0.73)
Monocytes ($\times 10^3/\mu l$), median (IQR)	0.17 (0.12-0.22)	0.12 (0.08-0.18)	0.15 (0.10-0.21)	0.08 (0.04-0.12)	0.08 (0.04-0.12)
Neutrophils ($\times 10^3/\mu l$), median (IQR)	1.84 (0.86-2.72)	1.48 (0.86-2.37)	1.63 (0.9 -2.63)	1.32 (0.84-2.20)	1.57 (1.28-4.17)
NLR, median (IQR)	1.17 (0.83-1.50)	2.19 (1.31-3.75)	1.80 (1.12-3.30)	2.32 (1.55-3.96)	2.55 (93-265)
Platelets ($\times 10^3/\mu l$), median (IQR)	140-340 [a]	192 (140-247)	202 (179-232)	174 (102-261)	167 (93-265)
Hemoglobin (g/dl), median (IQR)	13.5-17.0 [a] (male)<br>11.5-15.0 [a] (female)	11.6 (6.5-12.9)	12.8 (11.8-12.9)	10.9 (9.6-12.5)	9.8 (8.6-11.4)
C3 (mg/dl), median (IQR)	86-160 [a]	59.5 (37.5-85.8)	84.0 (79.0-98.3)	53.5 (31.8-78.5)	43.0 (31.0-76.0)
C4 (mg/dl), median (IQR)	17-45 [a]	9.0 (4.0-19.0)	17.0 (12.0-20.8)	8.0 (4.0-19.0)	6.0 (4.0-18.0)
CH50 (U/ml), median (IQR)	25.0-48.0 [a]	34.6 (16.4-47.6)	47.7 (36.6-58.3)	24.2 (12.7-39.2)	24.2 (11.9-37.2)
C1q (μg/m), median (IQR)	<3 [a]	0.0 (0.0 - 5.6)	0.0 (0.0-0.0)	2.2 (0.0-9.7)	2.4 (0.0-10.6)
Medications					
Medication-naïve, *n*	—	27	0	27	27
Prednisone, *n*	—	48	23	25	0
Prednisone (mg/day), median (IQR)	—	2.0 (0.0-5.5)	2.5 (1.5-4.5)	0.0 (0.0-7.6)	0.0 (0.0-0.0)
Hydroxychloroquine, *n*	—	5	3	2	0
Immunosuppressants [b]	—	31	17	14	0

[a] Normal range

[b] Tacrolimus, ciclosporin, azathioprine, mycophenolate mofetil, and mizorbine.

NLR, neutrophil/lymphocyte ratio

Supplementary Table 4. Clinical characteristics of patients with RA, p-SS, AAV, or IgG4-RD in this study.

Clinical characteristic	RA (*n*=46)	p-SS (*n*=16)	AAV (*n*=27)	IgG4-RD (*n*=37)
Age (years), median (IQR)	45.0 (16.0-58.0)	63.0 (52.5-70.8)	71.0 (60.0-80.0)	58.0 (47.0-70.0)
Female, *n* (%)	40 (87.0)	16 (100)	12 (44.0)	17 (47.2)
White blood cells ($\times 10^3$/μl), median (IQR)	6.35 (5.48-7.90)	4.95 (3.58-6.13)	8.60 (6.80-13.6)	6.00 (4.65-6.95)
Lymphocytes ($\times 10^3$/μl), median (IQR)	1.24 (0.91-1.54)	1.31 (0.99-1.73)	1.18 (0.84-1.41)	1.40 (1.02-1.69)
Monocytes ($\times 10^3$/μl), median (IQR)	0.20 (0.15-0.27)	0.17 (0.12-0.20)	0.20 (0.13-0.29)	0.21 (0.12-0.29)
Neutrophils ($\times 10^3$/μl), median (IQR)	3.12 (1.56-3.72)	1.75 (1.48-2.32)	3.89 (2.24-7.34)	1.82 (1.38-2.85)
NLR, median (IQR)	2.17 (1.48-3.11)	1.31 (0.97-1.74)	4.12 (1.59-6.66)	1.54 (1.02-1.98)
CRP (mg/dl), median (IQR)	0.9 (0.2-2.7)	0.1 (0.0-0.2)	5.5 (0.7-8.8)	0.1 (0.0-0.2)
Disease activity, median (IQR)	DAS28:<br>5.0 (4.0-6.0)	ESSDAI:<br>1 (0-4)	BVAS:<br>12 (9-18)	IgG4-RD RI:<br>12 (9-14)
Autoantibody-positive, *n* (%)	RF: 31 (67.4)<br>ACPA: 31 (67.4)	Anti-SS-A: 11 (68.8)<br>Anti-SS-B: 8 (50.0)	Anti-PR3: 5 (18.5)<br>Anti-MPO: 22 (81.5)	—<br>—
IgG4 (mg/dl), median (IQR)	—	—	—	375 (274-6245)
IgG (mg/dl), median (IQR)	1471 (1194-1784)	1565 (938-2319)	1800 (1356- 2027)	1723 (1449-2100)

NLR, neutrophil/lymphocyte ratio; RF, rheumatoid factor; ACPA, anti-citrullinated protein antibodies; ANCA, antineutrophil cytoplasmic antibody; DAS, disease activity score; ESSDAI, EULAR Sjögren's syndrome disease activity index; BVAS, Birmingham vasculitis activity score; IgG4-RD RI, IgG4-related disease responder index

Supplementary Table 5. Cell number of Tph subsets of patients with autoimmune diseases.

Cells	Cell number (cells/μl), median (IQR)		Cell number (cells/μl), median (IQR)				
	HC (*n*=33)	SLE (*n*=85)	Untreated SLE (*n*=27)	Untreated RA (*n*=46)	Untreated p-SS (*n*=16)	Untreated AAV (*n*=27)	Untreated IgG4-RD (*n*=37)
Tph	3.55 (2.28-5.28)	3.70 (2.50-6.45) ns	1.75 (0.25-3.45)	6.28 (4.68-8.96) **	5.78 (3.21-9.28) ns	5.55 (3.60-8.10) ns	7.90 (6.08 – 12.28) ****
Tph1	2.25 (1.23-3.80)	2.60 (1.55-4.43) ns	2.60 (0.85-4.65)	3.90 (2.89-5.23) *	3.01 (1.53-5.21) ns	3.50 (2.20-4.70) ns	4.40 (3.18 – 6.80) **
Tph2	0.15 (0.55-0.25)	0.25 (0.10-0.53) ##	0.30 (0.10-0.70)	0.30 (0.15-0.65) ns	0.28 (0.21-0.49) ns	0.55 (0.25-0.85) ns	1.30 (0.60 – 3.13) ****
Tph17	0.25 (0.20-0.50)	0.15 (0.05-0.30) ##	0.18 (0.04-0.31)	0.45 (0.20-0.71) ***	0.35 (0.16-0.49) **	0.45 (0.25-0.75) ***	0.40 (0.30 – 0.65) ****
Tph1-17	0.70 (0.55-0.98)	0.60 (0.35-1.25) ns	0.55 (0.30-1.05)	1.38 (0.89-2.59) ****	2.10 (1.15-2.80) ****	0.80 (0.50-1.35) *	0.85 (0.63 – 1.48) *

Data represent the median (IQR) of cell numbers (cells/μl) in each cell population.
Statistical difference between SLE and HC was analyzed by Mann-Whitney *U* test. ##*P*<0.01, ns: not significant.
Statistical difference between untreated SLE and RA, p-SS, AAV, or IgG4-RD was analyzed by Mann-Whitney *U* test. **P*<0.05, ***P*<0.01, ****P*<0.001, *****P*<0.0001, ns: not significant.

Supplementary Table 6. The correlation between Tph subsets and NLR in SLE patients.

Cells	Spearman correlation with NLR	
	r value	*P* value
Tph	0.24	0.0277 *
Tph1	0.24	0.0262 *
Tph2	0.22	0.0420 *
Tph17	0.06	0.6775 ns
Tph1-17	0.19	0.0788 ns

NLR, neutrophil/lymphocyte ratio
The correlation between the frequency of Tph subsets and NLR was analyzed by Spearman's rank correlation coefficients in SLE patients (n=85). *P<0.05. ns: not significant.

Supplementary Table 7. Cell number of Tph subsets in the blood of SLE patients with various clinical manifestations.

Cells	Cell number (cells/μl), median (IQR)					
	Nephritis		Cutaneous		Musculoskeletal	
	Without (*n*=51)	With (*n*=34)	Without (*n*=58)	With (*n*=27)	Without (*n*=63)	With (*n*=22)
Tph	4.05 (2.70-7.85)	3.28 (2.41-5.83) ns	3.68 (2.60-6.05)	4.85 (1.75-6.50) ns	3.90 (2.60-6.20)	3.53 (2.01-6.83) ns
Tph1	2.75 (1.75-4.50)	2.33 (1.23-3.93) ns	2.60 (1.64-4.36)	3.25 (0.95-4.80) ns	2.60 (1.64-4.46)	3.25 (0.95-4.80) ns
Tph2	0.25 (0.10-0.40)	0.35 (0.10-0.61) ns	0.25 (0.10-0.43)	0.35 (0.10-0.65) ns	0.25 (0.10-0.50)	0.25 (0.10-0.63) ns
Tph17	0.20 (0.05-0.35)	0.13 (0.00-0.30) ns	0.15 (0.05-0.32)	0.15 (0.10-0.30) ns	0.20 (0.05-0.35)	0.10 (0.05-0.30) ns
Tph1-17	0.75 (0.35-1.35)	0.55 (0.30-1.10) ns	0.58 (0.35-1.25)	0.75 (0.25-1.30) ns	0.65 (0.35-1.25)	0.55 (0.34-1.31) ns

Cells	Serositis		Fever		Alopecia	
	Without (*n*=72)	With (*n*=13)	Without (*n*=72)	With (*n*=13)	Without (*n*=75)	With (*n*=10)
Tph	3.95 (2.60-6.35)	3.40 (1.25-6.70) ns	4.03 (2.66-6.50)	2.60 (0.85-6.15) ns	3.90 (2.60-6.40)	3.10 (1.45-6.83) ns
Tph1	2.65 (1.61-4.39)	2.50 (0.95-5.13) ns	2.70 (1.75-4.44)	1.60 (0.45-4.13) ns	2.60 (1.65-4.40)	2.20 (0.76-4.73) ns
Tph2	0.25 (0.10-0.50)	0.35 (0.05-0.95) ns	0.25 (0.10-0.55)	0.25 (0.08-0.40) ns	0.25 (0.10-0.50)	0.38 (0.08-0.68) ns
Tph17	0.15 (0.05-0.34)	0.15 (0.00-0.28) ns	0.18 (0.05-0.34)	0.10 (0.00-0.28) ns	0.15 (0.05-0.30)	0.25 (0.08-0.41) ns
Tph1-17	0.70 (0.36-1.29)	0.55 (0.28-0.73) ns	0.65 (0.40-1.25)	0.30 (0.23-1.05) ns	0.60 (0.35-1.25)	0.35 (0.20-0.83) ns

Cells	Neuropsychiatric		Respiratory	
	Without (*n*=77)	With (*n*=8)	Without (*n*=77)	With (*n*=8)
Tph	3.70 (2.58-6.35)	4.00 (1.91-6.70) ns	4.00 (2.60-6.45)	2.63 (1.91-5.73) ns
Tph1	2.60 (1.55-4.33)	3.08 (1.05-4.76) ns	2.70 (1.63-4.43)	1.83 (1.03-4.60) ns
Tph2	0.25 (0.10-0.55)	0.18 (0.05-0.39) ns	0.25 (0.10-0.53)	0.38 (0.06-0.53) ns
Tph17	0.15 (0.05-0.30)	0.05 (0.01-0.44) ns	0.15 (0.05-0.33)	0.13 (0.01-0.28) ns
Tph1-17	0.60 (0.35-1.25)	0.70 (0.30-1.28) ns	0.70 (0.35-1.28)	0.38 (0.26-0.63) *

Data represent the median (IQR) of cell numbers (cells/μl) in each cell population.
Statistical difference between with and without manifestation was analyzed by Mann-Whitney *U* test. **P*<0.05 ns: not significant.

Supplementary Table 8. Cell number of each Tph subset before and the time achieved LLDAS in SLE patients with various clinical manifestations.

Cells	Cell number (cells/μl), median (IQR)					
	Nephritis (*n*=17)		Cutaneous (*n*=14)		Musculoskeletal (*n*=11)	
	Pre	LLDAS	Pre	LLDAS	Pre	LLDAS
Tph	4.85 (2.58-6.20)	2.80 (2.13-3.83) ns	5.48 (2.24-9.05)	2.98 (2.10-3.83) *	2.65 (1.20-6.90)	2.35 (1.60-4.05) ns
Tph1	2.70 (1.70-4.63)	1.65 (1.43-2.63) ns	3.53 (1.53-6.21)	1.68 (1.25-2.56) *	2.05 (0.50-5.00)	1.40 (1.05-2.75) ns
Tph2	0.30 (0.05-0.78)	0.15 (0.10-0.28) ns	0.35 (0.05-0.96)	0.13 (0.05-0.36) *	0.25 (0.05-1.05)	0.10 (0.05-0.25) ns
Tph17	0.10 (0.00-0.25)	0.30 (0.15-0.38) ns	0.15 (0.09-0.40)	0.44 (0.25-0.44) ns	0.10 (0.00-0.20)	0.20 (0.10-0.30) ns
Tph1-17	0.73 (0.40-1.10)	0.53 (0.32-0.79) ns	0.95 (0.33-1.19)	0.63 (0.34-1.24) ns	0.55 (0.25-0.75)	0.40 (0.15-0.90) ns

Data represent the median (IQR) of cell numbers (cells/μl) in each cell population.
Statistical difference between pre and LLDAS was analyzed by Wilcoxon's signed-rank test. *$P<0.05$, ns: not significant.

Supplementary Table 9. Cell number of each Tph subset in the patients with inadequate response for the medications.

Cells	Cell number (cells/μl), median (IQR)					
	Nephritis (*n*=5)		Cutaneous (*n*=4)		Musculoskeletal (*n*=3)	
	Pre	12M	Pre	12M	Pre	12M
Tph	3.38 (3.05-10.50)	2.85 (0.95-3.66) ns	3.93 (1.60-5.76)	3.43 (2.70-7.23) ns	3.40 (2.60-4.45)	2.65 (1.10-2.85) ns
Tph1	2.43 (1.58-7.81)	1.33 (0.53-2.14) *	2.92 (0.93-3.69)	2.13 (1.36-4.99) ns	2.50 (1.60-3.35)	1.15 (0.55-2.00) ns
Tph2	0.43 (0.33-0.71)	0.18 (0.00-0.48) ns	0.35 (0.14-0.49)	0.25 (0.08-0.65) ns	0.45 (0.25-0.60)	0.15 (0.00-0.35) ns
Tph17	0.15 (0.10-0.34)	0.33 (0.08-0.59) ns	0.20 (0.11-0.29)	0.40 (0.28-0.53) ns	0.10 (0.10-0.15)	0.35 (0.10-0.55) ns
Tph1-17	0.43 (0.30-2.06)	0.53 (0.16-0.93) ns	0.53 (0.26-1.39)	0.93 (0.41-1.36) ns	0.30 (0.30-0.75)	0.45 (0.35-0.60) ns

Data represent the median (IQR) of cell numbers (cells/μl) in each cell population.
Statistical difference between pre and post 12 months (M) was analyzed by Wilcoxon's signed-rank test. *P<0.05, ns: not significant.
