# Supplementary material for: Cytotoxic Tph subset with low B-cell helper functions and its involvement in systemic lupus erythematosus
Source: Commun Biol. 2024 Mar 6;7:277. doi: 10.1038/s42003-024-05989-x (PMC10918188; doi:10.1038/s42003-024-05989-x)
Supplement: Supplementary file 3 — Description of Additional Supplementary Files [file 42003_2024_5989_MOESM3_ESM.pdf]

Description of Additional Supplementary Files

File name: Supplementary Data 1

Description: The source data underlying the graphs in the article and supplementary information.
