# Supplementary material for: Cytotoxic Tph subset with low B-cell helper functions and its involvement in systemic lupus erythematosus
Source: Commun Biol. 2024 Mar 6;7:277. doi: 10.1038/s42003-024-05989-x (PMC10918188; doi:10.1038/s42003-024-05989-x)
Supplement: Supplementary file 5 — Reporting Summary [file 42003_2024_5989_MOESM5_ESM.pdf]

nature portfolio

Corresponding author(s): Kenji Chiba

Last updated by author(s): Dec 7, 2023

Reporting Summary

Nature Portfolio wishes to improve the reproducibility of the work that we publish. This form provides structure for consistency and transparency in reporting. For further information on Nature Portfolio policies, see our Editorial Policies and the Editorial Policy Checklist.

Statistics

For all statistical analyses, confirm that the following items are present in the figure legend, table legend, main text, or Methods section.

n/a	Confirmed	
☐	☒	The exact sample size (n) for each experimental group/condition, given as a discrete number and unit of measurement
☐	☒	A statement on whether measurements were taken from distinct samples or whether the same sample was measured repeatedly
☐	☒	The statistical test(s) used AND whether they are one- or two-sided<br>*Only common tests should be described solely by name; describe more complex techniques in the Methods section.*
☐	☒	A description of all covariates tested
☐	☒	A description of any assumptions or corrections, such as tests of normality and adjustment for multiple comparisons
☐	☒	A full description of the statistical parameters including central tendency (e.g. means) or other basic estimates (e.g. regression coefficient) AND variation (e.g. standard deviation) or associated estimates of uncertainty (e.g. confidence intervals)
☐	☒	For null hypothesis testing, the test statistic (e.g. F, t, r) with confidence intervals, effect sizes, degrees of freedom and P value noted<br>*Give P values as exact values whenever suitable.*
☒	☐	For Bayesian analysis, information on the choice of priors and Markov chain Monte Carlo settings
☒	☐	For hierarchical and complex designs, identification of the appropriate level for tests and full reporting of outcomes
☐	☒	Estimates of effect sizes (e.g. Cohen's d, Pearson's r), indicating how they were calculated

Our web collection on statistics for biologists contains articles on many of the points above.

Software and code

Policy information about availability of computer code

Data collection	Flow cytometriy analysis and cell sorting were performed by BD LSR Fortessa X-20 (BD biosciences) and BD FACS Aria II (BD biosciences), respectively. The ECL assay was performed using MESO QuickPlex SQ 120 (Meso Scale Discovery). NovaSeq 6000 instrument (Illumine) was used for RNA sequencing.
Data analysis	FACSDiva Software version 8.0.1 (BD biosciences) and FlowJo ver.10 (TreeStar) was use for flow cytometric analysis. The ECL assay and CBA assay was analyzed by Discovery Workbench 4.0.12 (MSD) and FCAP Array version 3.0.19.2091 (BD biosciences), respectively. The RNA sequence reads were aligned to the human reference genome (GRCh38) using Hisat2 version 2.2.1. Subsequently, mapped reads were analyzed with the HTSeq version 0.6.1 software for estimating the expression of each gene. Statistical analyses performed using GraphPad Prism software version 9.4.1 (GraphPad Prism Software Corporation)

For manuscripts utilizing custom algorithms or software that are central to the research but not yet described in published literature, software must be made available to editors and reviewers. We strongly encourage code deposition in a community repository (e.g. GitHub). See the Nature Portfolio guidelines for submitting code & software for further information.

Data

Policy information about availability of data

All manuscripts must include a data availability statement. This statement should provide the following information, where applicable:

- Accession codes, unique identifiers, or web links for publicly available datasets
- A description of any restrictions on data availability
- For clinical datasets or third party data, please ensure that the statement adheres to our policy

All data in this study are provided in the Supplementary Information.

Research involving human participants, their data, or biological material

Policy information about studies with human participants or human data. See also policy information about sex, gender (identity/presentation), and sexual orientation and race, ethnicity and racism.

Reporting on sex and gender	Information on gender of patients and healthy controls (HCs) were collected but sex differences were not analyzed .
Reporting on race, ethnicity, or other socially relevant groupings	Information on race, ethnicity, or other socially relevant groupings were not collected from patients and HCs.
Population characteristics	Patient details are listed in Supplementary Tables 3 and 4.
Recruitment	Patients with SLE (n=85), RA (n=46), p-SS (n=16), AAV (n=27), and IgG4-RD (n=37) were visited Keio University Hospital from May 2015–December 2019. HCs participated in this study were also recruited Keio University Hospital. We have confirmed that HCs did not have autoimmune disease, severe allergic disorder, malignancy or infection. Blood samples of patients were collected after written informed consent.
Ethics oversight	Ethical approval for this study was granted by the ethics committee of Keio University School of Medicine (protocol #20140335) and by Mitsubishi Tanabe Pharma Corporation (protocol #H14018).

Note that full information on the approval of the study protocol must also be provided in the manuscript.

Field-specific reporting

Please select the one below that is the best fit for your research. If you are not sure, read the appropriate sections before making your selection.

☒ Life sciences ☐ Behavioural & social sciences ☐ Ecological, evolutionary & environmental sciences

For a reference copy of the document with all sections, see nature.com/documents/nr-reporting-summary-flat.pdf

Life sciences study design

All studies must disclose on these points even when the disclosure is negative.

Sample size	Sample size was not determined before the start of this examination. Sample size in this study depended on sample recruitment at Keio University Hospital.
Data exclusions	No data was excluded in the analyses.
Replication	All experiments were repeated at least three times and similar results were observed in all experiments. The final data shown in the this study are the combination of all the experiments.
Randomization	Randomization was not conducted in this study. We used samples from patients who were diagnosed with SLE, RA, p-SS, AAV, or IgG4-RD according to their respective classification criteria at Keio University Hospital between May 2015 and December 2019. HCs were selected by random participants.
Blinding	In experiments, investigators were blinded to the clinical character and laboratory data of patients.

Reporting for specific materials, systems and methods

We require information from authors about some types of materials, experimental systems and methods used in many studies. Here, indicate whether each material, system or method listed is relevant to your study. If you are not sure if a list item applies to your research, read the appropriate section before selecting a response.

Materials & experimental systems

n/a	Involved in the study
☐	☒ Antibodies
☒	☐ Eukaryotic cell lines
☒	☐ Palaeontology and archaeology
☒	☐ Animals and other organisms
☐	☒ Clinical data
☒	☐ Dual use research of concern
☒	☐ Plants

Methods

n/a	Involved in the study
☒	☐ ChIP-seq
☐	☒ Flow cytometry
☒	☐ MRI-based neuroimaging

Antibodies

Antibodies used	FITC-conjugated anti-CD45RA antibody (Clone: HI100 Cat# 555488)<br>BV786-conjugated anti-CD3ε antibody (Clone: SK7, BD Biosciences, Cat# 563800)<br>PE-CF594-conjugated anti-CD3ε antibody (Clone: UCHT1, BD Biosciences, Cat# 562280)<br>PerCP-Cy5.5-conjugated anti-CD19 antibody (Clone:HIB19, BD Biosciences, Cat# 561295)<br>BUV395-conjugated anti-CD19 antibody (Clone:HIB19, BD Biosciences, Cat# 740287)<br>PE-Cy7-conjugated anti-CD4 antibody (Clone: SK3, BD Biosciences, Cat# 557852)<br>BUV737-conjugated anti-CD4 antibody (Clone: SK3, BD Biosciences, Cat# 612789)<br>BUV563-conjugated anti-CD8 antibody (Clone: RPA-T8, BD Biosciences, Cat# 612914)<br>BV510-conjugated anti-PD-1 antibody (Clone: EH12.1, BD Biosciences, Cat# 563076)<br>PE-conjugated anti-CXCR3 antibody (Clone: 1C6/CXCR3, BD Biosciences, Cat# 557185)<br>BV421-conjugated anti-CXCR5 antibody (Clone: RF8B2, BD Biosciences, Cat# 562747)<br>PE/Cy7-conjugated anti-CCR5 antibody (Clone: 2D7, BD Biosciences, Cat# 557752<br>BV711-conjugated anti-CXCR3 antibody (Clone: G025H7, BioLegend, Cat# 353732)<br>PerCP/Cy5.5-conjugated anti-CCR6 antibody (Clone: G034E3, BioLegend, Cat# 353406)<br>APC-conjugated anti-CCR6 antibody (Clone: G034E3, BioLegend, Cat# 353416)<br>APC/Cy7-conjugated anti-CCR6 antibody (Clone: G034E3, BioLegend, Cat# 353432)<br>PE/Dazzle594-conjugated anti-CCR2 antibody (Clone: K036C2, BioLegend, Cat# 357222)<br>APC-conjugated anti-CCR2 antibody (Clone: K036C2, BioLegend, Cat# XXX)<br>PE-conjugated anti-CX3CR1 antibody (Clone: 2A9-1, BioLegend, Cat# 341604)<br>APC-conjugated anti-perforin antibody (Clone: dG9, BioLegend, Cat# 308112)<br>Pacific blue-conjugated anti-granzyme B antibody (Clone: GB11, BioLegend, Cat# 515408)
Validation	Antibodies were validated by their manufacturers and used according to the manufacturer's instruction. Furthermore, antibodies have been re-validated by titrating their concentration. Isotypes and FMO were included in the experiments whenever appropriate.

Clinical data

Policy information about clinical studies

All manuscripts should comply with the ICMJE guidelines for publication of clinical research and a completed CONSORT checklist must be included with all submissions.

Clinical trial registration	n/a
Study protocol	n/a
Data collection	n/a
Outcomes	n/a

Flow Cytometry

Plots

Confirm that:

☐ The axis labels state the marker and fluorochrome used (e.g. CD4-FITC).

☒ The axis scales are clearly visible. Include numbers along axes only for bottom left plot of group (a 'group' is an analysis of identical markers).

☐ All plots are contour plots with outliers or pseudocolor plots.

☒ A numerical value for number of cells or percentage (with statistics) is provided.

Methodology

Sample preparation	Cell surface maker staining: Heparinized blood samples were collected from patients or HCs. Blood samples were stained

Sample preparation	with appropriate monoclonal antibodies (mAbs) and fixed by Phosflow Lyse/Fix Buffer.<br><br>Intracellular staining: Blood samples were stained with anti-cell surface maker mAbs. The cells were fixed with 4% paraformaldehyde and permeabilized with 0.5% Triton X-100. After blocking with 3% BSA-PBS, the cells were stained with anti-granzyme B and anti-perforin mAbs.<br><br>PBMCs separation for Tph cells sorting: Peripheral blood mononuclear cells (PBMCs) were obtained by density gradient centrifugation using Ficoll. PBMCs were staining with anti-CD3, CD4, CD8, CD19, CD45RA, PD-1, CXCR3, CXCR5, and CCR6 mAbs.
Instrument	BD LSRFortessa X-20<br>BD FACS Aria II
Software	BD FACS Diva Software version 8.0.1<br>FlowJo ver.10
Cell population abundance	Post-sort cell populations were generally > 95% pure as determined by flow cytometry.
Gating strategy	Figure 1a showed the gating strategy of Tph subsets.<br>The initial gating used FSS-A/SCC-A gating to gate leukocytes and to exclude debris. Then, we determined PD-1hiCXCR5- Tph cells within CD45RA- memory CD4+ T cells and subsequently defined 4 Tph subsets based on the cell surface expressions of CXCR3 and CCR6. Tph subsets were determined as follows: Tph1, CXCR3+CCR6-; Tph2, CXCR3-CCR6-; Tph17, CXCR3-CCR6+; Tph1-17, CXCR3+CCR6+ based on the expression of CXCR3 and CCR6.

☒ Tick this box to confirm that a figure exemplifying the gating strategy is provided in the Supplementary Information.
